# Role of the Autonomic Nervous System in Mechanism of Energy and Glucose Regulation Post Bariatric Surgery

**DOI:** 10.3389/fnins.2021.770690

**Published:** 2021-11-23

**Authors:** Zhibo An, Haiying Wang, Mohamad Mokadem

**Affiliations:** ^1^Division of Gastroenterology and Hepatology, Department of Internal Medicine, Carver College of Medicine, The University of Iowa, Iowa City, IA, United States; ^2^Department of Physiology, Basic Medical School of Jining Medical University, Jining, China; ^3^Fraternal Order of Eagles Diabetes Research Center, The University of Iowa, Iowa City, IA, United States; ^4^Obesity Research and Education Initiative, The University of Iowa, Iowa City, IA, United States; ^5^Iowa City Veterans Affairs Health Care System, Iowa City, IA, United States

**Keywords:** parasympathetic nervous system, sympathetic nervous system, energy balance, metabolic regulation, bariatric/metabolic surgery

## Abstract

Even though lifestyle changes are the mainstay approach to address obesity, Sleeve gastrectomy (SG) and Roux-en-Y gastric bypass (RYGB) are the most effective and durable treatments facing this pandemic and its associated metabolic conditions. The traditional classifications of bariatric surgeries labeled them as “restrictive,” “malabsorptive,” or “mixed” types of procedures depending on the anatomical rearrangement of each one of them. This conventional categorization of bariatric surgeries assumed that the “restrictive” procedures induce their weight loss and metabolic effects by reducing gastric content and therefore having a smaller reservoir. Similarly, the “malabsorptive” procedures were thought to induce their main energy homeostatic effects from fecal calorie loss due to intestinal malabsorption. Observational data from human subjects and several studies from rodent models of bariatric surgery showed that neither of those concepts is completely true, at least in explaining the multiple metabolic changes and the alteration in energy balance that those two surgeries induce. Rather, neuro-hormonal mechanisms have been postulated to underly the physiologic effects of those two most performed bariatric procedures. In this review, we go over the role the autonomic nervous system plays- through its parasympathetic and sympathetic branches- in regulating weight balance and glucose homeostasis after SG and RYGB.

## Introduction

Obesity is associated with several co-morbidities that carry a significant burden on the healthcare system and quality of life of all affected subjects around the world. As of 2014, 13% of adults worldwide were obese, with the most affected region being the American continent, for the prevalence of obesity among adults in the United States increased from 33.7% in 2007–2008 to 42.4% in 2017–2018 (Hales et al., 2020). Prediction models suggest that the prevalence of overweight condition in the United States will surpass 75% and that of obesity will be ∼50% by the year of 2030 (Wang et al., 2011). The initial and conventional approach for managing obesity involves multiple lifestyle modifications that implement changes in diet, activity and behavior. However, these measures rarely result in durable or drastic weight loss (Wadden et al., 2012). In the face of this raging epidemic, bariatric and metabolic surgery remains the most effective intervention to manage excess body weight and adiposity and it significantly improves most obesity-related morbidities with results lasting more than a decade. Needless to say, because of its invasiveness as well as short and long-term complications (though significantly improved) it is currently offered for selected patients who are morbidly obese and who meet certain specific criteria, after failing conventional therapy. Several types of bariatric surgeries including SG, RYGB, and other forms of gastric bypass, laparoscopic adjustable gastric banding, and duodenal switch with biliopancreatic diversion, are nowadays the most common surgical procedures being offered to patients with obesity (English et al., 2020). Though RYGB was traditionally considered by many to be the gold standard weight loss procedure, SG had become the most commonly performed bariatric surgery since 2013 after its reimbursement was approved by Medicare and Medicaid in 2012 (English et al., 2018). It has been demonstrated that sleeve and RYGB have comparable weight loss outcomes, at least in the short-term (Schauer et al., 2012).

The traditional theory which attributes “restriction” of gastric pouch to reduced caloric consumption and small intestinal bypass to decreased caloric absorption has been repetitively evaluated. It was shown that gastric pouch or sleeve size did not affect meal size or food intake reduction post-bariatric surgery (Topart et al., 2011; McCracken et al., 2018). Additionally, the amount or caloric malabsorption induced by gastric bypass was very modest and cannot explain solely or be responsible for the large amount of weight lost induced by this bariatric procedure (Odstrcil et al., 2010). On the other hand, it was previously shown that alteration in feeding behaviors, bile acid signaling and flow, gut microbiota, as well as several neuro-hormonal effects (through GLP-1, P-YY, Ghrelin, and leptin) play an important role in inducing many of the beneficial effects of bariatric surgery (le Roux et al., 2007; Stefater et al., 2012; Liou et al., 2013; Yan et al., 2014). Obesity was previously reported to be associated with an attenuated vagal tone and a selective increase in sympathetic tone activity specifically to musculoskeletal organs and peripheral arterial bed, predisposing to obesity-associated hypertension (Landsberg, 1986, 2001; Guarino et al., 2017). There is suggestive evidence that RYGB transiently activates the systemic sympathetic nervous system, leading to a subsequent relative enhancement in parasympathetic over sympathetic activity, that seemingly lasts longer (Lips et al., 2013). In addition, other studies suggested that the gut-brain communications via parasympathetic or sympathetic neuron fibers (afferent/sensory or efferent/motor) may also play a differential role in regulating metabolic effects of bariatric surgery (Berthoud, 2008; Ballsmider et al., 2015).

Both arms of the autonomic nervous system have been postulated to play a metabolic regulatory role in bariatric surgery. This review will review go over each system (sympathetic and parasympathetic) in regulating variable metabolic effects of RYGB and SG.

## Introduction of Sympathetic and Parasympathetic Nerves and Their Implications in Metabolism

The autonomic neurons of the sympathetic and parasympathetic nervous system innervate multiple organs and regulate their many of their homeostatic functions. The autonomic nervous system often works in a closed-loop feedback mechanism where sensing of variable biological factors is transmitted via afferent neurons to the brain or brain stem. Subsequently, homeostatic centers or nuclei within the central nervous system process these signals and transmit response orders via efferent/motor neurons to target body organs. Both sympathetic and parasympathetic nervous systems maintain whole-body homeostasis (e.g., through their actions on circulation, energy metabolism, thermal situation, respiration, and immunity) in the face of both endogenous and exogenous perturbations (Esler et al., 2003).

An earlier hypothesis postulated that weight gain in obesity is partially due to sympathetic nervous underactivity causing reduction in thermogenesis (Tremblay and Chaput, 2009). However, microneurography and regional noradrenaline spillover measurements in obese individuals have disproven this hypothesis, thus weakening the case for therapeutic use of β3-adrenergic agonists to stimulate thermogenesis (Kamiya et al., 2021). Interestingly, weight loss, secondary to lifestyle interventions such as diet and exercise, improves sympathetic and parasympathetic heart rate variability, which is an index of autonomic control of cardiac activity. Bariatric surgery also improves HRV through weight loss which might also prevent cardiac autonomic neuropathy (CAN) in severe obesity (Williams et al., 2019).

The peripheral nervous system is also involved in direct and indirect regulation of food intake. Vagal afferent neurons in the duodenum and stomach are shown to respond to mechanical stretch by luminal nutrients while also integrating other visceral sensory information along with metabolic, signals, through neuronal projection into brain stem centers. After vagotomy, neuroendocrine signaling pathways from gut hormones, such as ghrelin, are disrupted. Although, celiac branch vagotomy performed concomitantly with RYGB in rat model resulted in a slightly lesser degrees of weight reduction compared to RYGB without vagotomy, this effect seemed to be transient (Hao et al., 2014). Weight loss outcome in human subjects who had RYGB did not seem to be affected by having a vagotomy or not (Okafor et al., 2015). This data suggest that vagal contribution to RYGB-induced weight loss- if present- is already induced at time of surgery, supporting in part the Phantom satiation hypothesis by Gautron (2021). Tracer studies showed that RYGB decreases the density of vagal sensory neurons and also activates microglia in the NTS, altering neuronal communication – and assumingly energy signaling- between the gut and the CNS (Mulla et al., 2018).

## Laparoscopic Sleeve Gastrectomy and Roux-En-Y Gastric Bypass

### Weight Loss

Roux-en-Y gastric bypass, traditionally considered as the gold standard weight loss procedure, has become the second most performed bariatric procedure since 2013. Laparoscopic sleeve gastrectomy (LSG) has become the most performed bariatric procedure worldwide, because of its efficacy in achieving weight loss results and improvements in obesity-associated comorbidities that are comparable to those of RYGB with lower operative and post-operative complications (Rosenthal et al., 2012; Parikh et al., 2013). In comparison with other bariatric surgeries, such as the laparoscopic RYGB, LSG is a shorter and less technically challenging procedure that involves fewer changes in gastrointestinal anatomy. Additionally, the stomach is longitudinally dissected during LSG, with lesser disruption of the distal fibers of gastric vagus, whereas the stomach is transversely transected during RYGB and both the dorsal and the ventral branches of the gastric vagus nerve are disrupted, creating damage to preganglionic efferent and afferent fibers (Berthoud et al., 2011; Saeidi et al., 2012; Browning et al., 2013; Hao et al., 2014).

### Glucose Control

Bariatric surgery, including RYGB and LSG, markedly ameliorate glycemic control and may reverse or prevent T2DM in individuals with obesity (Mingrone et al., 2015; Schauer et al., 2017; Borgeraas et al., 2020; McTigue et al., 2020). The metabolic benefits appear to be weight independent as they occur prior to the onset of significant weight loss and are not correlated with weight loss magnitude. The exact underlying mechanisms are still unclear (Mingrone and Cummings, 2016). Multiple pathways have been proposed to be involved in the positive metabolic effects of RYGB, such as adipose function and morphology, glucose turnover in the liver, altered route and timing of food delivery to the small bowel, CNS control of metabolism and nutrient intake, as well as altered hormonal signaling (Batterham and Cummings, 2016). Following RYGB, there is a marked and rapid increase in several gut hormones including glucagon-like peptide-1 (GLP-1), and glucose-dependent insulinotropic peptide (GIP), specifically in the postprandial state- and it is thought the incretin effect of these hormones contributes to improved glycemic control (Falken et al., 2011; Jorgensen et al., 2012). There is also data suggesting that the incretin effect of GLP-1 is not regulated through endocrine pathway via beta cells, supporting the hypothesis that GLP-1 promotes insulin secretions via paracrine and/or neurocrine action (Smith et al., 2014). Rapid delivery to the distal small bowel with ingested nutrients is thought to contribute to the increased GLP-1 response (Salehi et al., 2011). The known incretin effect of GLP-1 along with its central glucoregulatory effects (Parlevliet et al., 2010) and its energy regulatory effect on food intake (Ten Kulve et al., 2017), may partially explain improved glucose profile following RYGB.

Comparing the early weight loss-independent and later weight loss-dependent (after 1 year) glycemic controls of these surgeries, LSG and RYGB showed similar changes on glycemic control, despite lower concentrations of GLP-1 and slightly less BMI reduction following LSG (Wallenius et al., 2018). This is in line with the observation in rodents that even complete absence of GLP-1 does not attenuate the LSG-mediated effects on weight reduction and glucose metabolism (Wilson-Perez et al., 2013).

The increased profile of GLP-1 after bariatric surgeries is thought to be explained by the hind-gut theory, in which stimulation of the distal intestine resulted from rapid nutrient entry further down the gastrointestinal tract such as ileum, where most of L-cells are found (Chambers et al., 2014). In addition, it was suggested that intestinal adaptation following chronic exposure to rapid nutrient delivery may lead to expansion in enteroendocrine cell population or nutrient-sensing capability, which contribute to the exaggerated increase in levels of postprandial GLP-1 after LSG or RYGB. It has to be noted that this observation is more consistent with RYGB than with LSG (Hutch and Sandoval, 2017). Interestingly, there were studies showing that LSG may lead to augmented elevation in postprandial GLP-1 (Ramon et al., 2012; Yousseif et al., 2014) comparable to that of RYGB.

Peptide YY (PYY), an anorexigenic peptide secreted by L-cells mostly located in the ileum and colon, reduces appetite, decreases the contraction of gallbladder, slows gastric emptying, suppresses gastric and pancreatic secretion, and increases nutrient absorption in the ileum. It was demonstrated that reduced PYY secretion in obese patients significantly increases after LSG, comparable to those observed after RYGB (Benaiges et al., 2015).

## Sympathetic Nervous System and Roux-En-Y Gastric Bypass

Bariatric surgery improves metabolic abnormalities in morbidly obese individuals. Hyperlipidemia, hypertension, T2DM, and obstructive sleep apnea are improved substantially following the surgery. Although improved blood pressure following RYGB has been attributed to weight-loss, lower blood pressure can occur before the reduction of body weight (Ahmed et al., 2009). Therefore, the blood pressure -lowering effect secondary to surgery may be weight loss independent. The potential underlying mechanisms of the blood pressure -lowering effects following RYGB include enhanced secretion of incretins, such as GLP-1 and PYY (Ochner et al., 2011), reducing leptin levels (Rodriguez et al., 2012), alerting microbiota in the GI tract (Lin et al., 2019), increasing excretion of urinary sodium (Docherty et al., 2017), and decreasing sympathetic nervous system activity (Zhang et al., 2014).

It is recognized that obesity is characterized by sympathetic nervous activation which contributes to hypertension associated with obesity. The time course of this change as well as the underlying mechanisms are not completely understood (Lohmeier and Iliescu, 2013). In addition, obesity and overweightness are characterized by sympathetic overactivity which mirrors the severity of the clinical condition and reflects metabolic alterations (Grassi et al., 2019). In the context of obesity, it has been hypothesized that increased resting sympathetic neural activity (tone) occurring following weight gain could be an adaptive mechanism to increase resting energy expenditure therefore reset the body weight back to a given set-point (Landsberg, 1986).

Using microneurography to directly measure sympathetic activity, it was demonstrated that human subjects who had undergone RYGB experience a reduced systemic sympathetic tone, specifically a muscular sympathetic nerve activity contributing (MSNA) to total energy expenditure (Curry et al., 2013).

Endocannabinoids, originally believed to be primarily central neuromodulators, can stimulate autonomic sympathetic pathway, regulate fat intake, and enhance energy expenditure (Quarta et al., 2010; DiPatrizio et al., 2011; Cardinal et al., 2014). Our recent *in vivo* studies with mice suggested that RYGB but not LSG increases splanchnic nerve activity, which induces thermogenesis of visceral fat and enhances resting metabolic rate (Ye et al., 2020). Furthermore, use of an endocannabinoid receptor-1 (CB1) inverse agonist, mirrors RYGB-specific effects on energy expenditure and gut’s sympathetic nerve activity. Whereas arachidonoylethanolamide, a CB1 agonist, attenuated the weight loss that was induced by RYGB. Therefore, this “browning” of visceral fat post-RYGB – which is mediated by sympathetic nerve activity- appears to be CB1 signaling dependent. Our findings suggested that CB1 plays a pivotal role in energy balance following RYGB via a pathway that the sympathetic nervous system is involved (Ye et al., 2020).

In summary, obesity seems to be associated with a state of increased sympathetic tone activity (in particular MSNA). RYGB has shown to decrease systemic sympathetic nerve activity (SNA) and likely to increase splanchnic sympathetic nerve activity, selectively, to activate thermogenesis and lipolysis of visceral white adipose tissue.

## Parasympathetic Nervous System and Roux-En-Y Gastric Bypass

The vagus nerve can act on the stomach and affects weight loss. The gastric sensory input is conveyed to the CNS via gastric vagal afferents, the central terminals of which enter the brainstem via the solitary tract and synapses of neurons on the nucleus tractus solitarius (NTS) (Altschuler et al., 1989; Berthoud and Powley, 1992; Fox et al., 2000; Czaja et al., 2006). ∼70% of vagal afferents innervate the abdominal viscera, mostly the intestines and stomach (Prechtl and Powley, 1990; Berthoud and Powley, 1992; Powley and Phillips, 2002). Abdominal vagal afferent signaling is important in the regulation of food intake following gastrointestinal stimuli (Peters et al., 2005; Campos et al., 2012, 2013). Efferent innervation to the stomach originates from the dorsal motor nucleus of the vagus (Kirchgessner and Gershon, 1989; Berthoud et al., 1991; Moran et al., 1997), which then projects to the myenteric plexus, terminating in the stomach with the highest density of efferent nerves (Berthoud et al., 1991). Furthermore, NTS preganglionic neurons can control the cholinergic excitatory and non-adrenergic non-cholinergic (NANC) inhibitory postganglionic neurons (Broussard and Altschuler, 2000). *In vivo* studies showed that RYGB causes a significant reduction in the weight of rats with T2DM, augmented the concentrations of serum insulin and GLP-1. These metabolic effects following RYGB partially depend on hepatic branch of the vagus nerve, as selective vagotomy of this nerve is associated with weight regain and the relative lower levels of serum GLP-1 and insulin (Qiu et al., 2014).

Ballsmider et al. (2015) found that LSG upregulated, whereas RYGB downregulated the density of the vagal afferents on the NTS in rats. In addition, RYGB, but not LSG, significantly activated microglia in the NTS. These findings suggested that RYGB, but not LSG, leads to vagal microglia activation and remodels gut-brain actions (Ballsmider et al., 2015). In line with this, it was recently demonstrated that subdiaphragmatic vagotomy can remodel central vagal afferent terminals in the NTS (Peters et al., 2013), and activate microglia in the DMV, NTS, and nodose ganglia. This microglia remains markedly activated in the DMV and nodose ganglia for 7 weeks after subdiaphragmatic vagotomy (Gallaher et al., 2012). Based on this delineated role of the vagus nerve mediating gut function and relaying sensory input to feeding centers in the hindbrain, it was speculated that these processes are remodeled after bariatric procedure (Berthoud et al., 2011).

Melanocortin-4 receptors (MC4R) are expressed in the hypothalamus and hindbrain as well as in autonomic neurons including parasympathetic vagal sensory neurons and preganglionic cholinergic motor neurons (parasympathetic and sympathetic). They play an important role in regulation food intake and energy expenditure in response to peripheral energy signals such as micro-nutrients or gut hormones. In line with these observations, severe obesity was found in humans with naturally occurring *Mc4r* mutations and mice with *Mc4r-*deficiency (Huszar et al., 1997; Vaisse et al., 1998). Moreover, MC4Rs appear to be a mechanistic connection between the digestive system, CNS, and autonomic signaling to brown adipose tissue and the abdominal viscera (Zechner et al., 2013).

Furthermore, Zechner et al. (2013) found that MC4Rs in cholinergic preganglionic vagal motor neurons mediated glucose and lipid homeostasis improvements following RYGB and this effect was weight independent. While MC4R signaling in preganglionic cholinergic motor neurons (parasympathetic and sympathetic) is crucial for the increased energy expenditure and weight loss induced by RYGB (Zechner et al., 2013). A rare variant of carriers of MC4R named I251L, was shown to have enhanced weight loss following RYGB and augmented basal activity *in vitro*, as well as improved early diabetes resolution following surgery that is weight-independent than non-carriers. They suggested that MC4Rs mediated autonomic efferent signaling is key to induce metabolic effects following RYGB, including these weight-independent benefits such as improved glucose profile (Zechner et al., 2013).

An endogenous agonist of the peroxisome proliferator-associated receptor-α (PPAR-α), Oleoylethanolamide (OEA), is produced by enterocytes of the upper small intestine. It was shown that OEA can activate PPAR-α receptors via vagal sensory output to suppress fat intake through striatal (Tellez et al., 2013) and hypothalamic (Gaetani et al., 2010) feeding circuits. Hankir et al. (2017) demonstrated that RYGB stimulated lower small intestine production of OEA, and augmented lipid sensing in the gut through PPAR-α, which subsequently relayed this signal to the CNS via vagal afferents neurons. This vagal signal led to increased dorsal striatal dopamine 1 receptor (D1R) expression/and signaling (Hankir et al., 2017). It was suggested that fat consumption post-RYGB is dependent on local OEA, vagus nerve and dorsal striatal D1R signaling, as interfering with them reversed the metabolic benefits of RYGB on fat preferences and intake (Hankir et al., 2017).

Previous studies have shown a beneficial effect of weight loss primarily on measures of parasympathetic activity after RYGB (Maser et al., 2007; Perugini et al., 2010). Other studies suggested that changes in autonomic tone after RYGB could be secondary to a direct effect of weight loss (Wasmund et al., 2011). It has been previously shown that glucose homeostasis and insulin secretions could be mediated by autonomic nerve system via neurotransmitters and GI peptides, which is thought to be part of the brain-gut signaling pathway that regulate metabolic effects following RYGB. For example, insulin and glucagon release can be regulated by peptides and neurotransmitters released from neurons innervating the islets (Ahren et al., 2006; Sterl et al., 2016). Acetylcholine, pituitary adenylate cyclase activating polypeptide, vasoactive intestinal polypeptide, and gastrin releasing peptide released from parasympathetic neurons all enhance insulin secretion (Bradley et al., 2012). It was shown that patients with T2DM who underwent RYGB, and experienced rapid remission of their diabetes not only had increased actions of GI peptides, but also had increased heart rate variability, particularly the high-frequency component, suggesting an enhanced parasympathetic outflow after RYGB as it has been demonstrated previously (Boido et al., 2015; Katsogiannos et al., 2020). The results support involvement of neuro-hormonal mechanisms in the rapid improvement of glucose metabolism following RYGB in T2DM. This again supports the theory of changes in autonomic nervous system activity following RYGB in modulating the metabolic effects of this surgery.

In summary, there is convincing evidence that the parasympathetic nervous system activity – represented mainly by the vagus nerve- is modulated after RYGB. This alteration in vagal tone has been tightly connected to changes in several gut hormones and early improvement in glucose metabolism post-RYGB.

## Sympathetic Nervous System and Laparoscopic Sleeve Gastrectomy

Reduced vagal function and increased sympathetic activity were observed in obese subjects. The improvement in parasympathetic tone following LSG was evidenced by enhanced heart rate variability in women with obesity, as early as the first month after surgery (Ibacache et al., 2020). LSG has shown a greater effect on the parasympathetic tone than RYGB, probably because LSG preserves the vagal trunk at the lesser curvature of stomach (Geronikolou et al., 2017).

Using high-fat diet induced obese mice underwent sham or LSG surgery and implantation of radio telemeters, McGavigan et al. (2017) found LSG decreased blood pressure in LSG-operated mice compared with both sham-operated groups (*ad libitum* and pair feeding), which were associated with a body weight-independent reduction in hypothalamic PERK-mediated ER stress, inflammation of hypothalamus and sympathetic nervous system tone.

The sympathetic nerve system is a key regulator in the production of leptin by white fat. Leptin production is extremely reduced in mice exposed to a cold environment, when sympathetic stimulation of white fat was enhanced. This can also occur after administration of norepinephrine and isoproterenol. Thus sympathetic nerve excitement inhibits the synthesis of leptin (Rayner and Trayhurn, 2001). In contrast, use of methyltyrosine to interfere with catecholamine synthesis can increase leptin levels in experimental animals. The sympathetic nerve system not only regulates leptin production, but also modulates its effects. In a mouse model where, sympathetic nerves are chemically removed, the original leptin effects of increased blood sugar, insulin, and glucagon were altered when exogenous leptin was given to mice (Holzman et al., 1999; Palmen et al., 2001). It was suggested that LSG-induced weight loss results in profound sympathoinhibitor effects, accompanied by a significant and stable attenuation in leptin levels of plasma, while the improved insulin sensitivity was decayed with time regardless (Seravalle et al., 2014).

Laparoscopic sleeve gastrectomy surgery reduces stomach capacity and removes the fundus by excising the larger curvature of the stomach, thereby reducing the level of circulating ghrelin, which is predominantly secreted from the fundus and upper gastric body. As the level of ghrelin is reduced, people are prone to feeling full and reducing food intake, thereby reducing caloric intake (Benaiges et al., 2015). It can also increase insulin secretion to have anti-diabetogenic effects. In addition, the levels of the insulin-promoting hormone GLP-1 and the appetite-suppressing polypeptide PYY3-36 secreted by the gastrointestinal tract are increased after the operation, which promotes the secretion of insulin and increases cellular insulin sensitivity. In addition, LSG surgery regulates blood sugar and triglyceride levels, reduces the secretion of antibiotic peptides such as leptin or monocyte chemoattractant protein 1, and increases anti-inflammatory mediators such as adiponectin.

Sympathetic neuron-associated macrophages (SAMs) are subset of macrophages recently characterized and identified within the white adipose tissue and long the sympathetic fibers innervating fat cells. They express SLC6A2 transporter which is responsible for noradrenaline degradation and consequently decreasing SNS- mediated thermogenesis. SAMs have been observed to be elevated in state of obesity and eliminating them or their degradation enzyme (SCLC6A2) results in activated thermogenesis. We do not know yet, the effect of either RYGB or SG on SAMs level or activity within WAT (Larabee et al., 2020).

In summary, LSG seems to have an early systemic neuro-inhibitory sympathetic effect that might be responsible for the blood pressure, and leptin reduction effects. There is limited proof that LSG (like RYGB) can induce activation of selective autonomic sympathetic nerves or tracts (such as the splanchnic) or augment sympathetic-mediated thermogenesis.

Bariatric surgery might also change taste acuity an/or olfaction, and potentially influence food preference and caloric consumption, which in turn leads to weight loss. Heightened sensitivity to sweetness could also be altered by the increase in GLP-1, P-YY and changes in other regulators hormones (such as insulin, ghrelin and leptin) that occur post RYGB and LSG. Several gut hormones and their receptors are expressed within the taste buds themselves, suggesting a possible role in palatability in addition to their known metabolic functions (Mulla et al., 2018).

## Parasympathetic Nervous System and Laparoscopic Sleeve Gastrectomy

Variable gastrointestinal signaling inputs are thought to be involved in ingestion of food, which can directly- or indirectly- activate vagal afferent nerve endings in a predominantly paracrine fashion to induce gastric relaxation, and pancreatic exocrine secretion. Circulating neurohormones such as CCK and GLP-1 act directly at the brainstem to modulate vagal afferent and efferent activity in addition to their potential actions as neurotransmitters within these neurocircuits (Grayson et al., 2014). One of the hypotheses regarding resolution of T2DM following bariatric procedures (specifically LSG) has been attributed to changes in gut hormone and alteration in anatomy. The rearrangement of gastrointestinal anatomy enhances simultaneous increase of GLP-1, P-YY, adiponectin and post-prandial insulin, and reduction in leptin as a result of fat mass reduction (Borges Mde et al., 2015). Other hypotheses suggest that elevated levels of bile acid, diet-induced thermogenesis, altered gut microbiome, or even changes in energy balance (due to reduction in food intake) per say leads to weight loss and consequently improvement in glucose homeostasis (Pournaras and le Roux, 2013; Hao et al., 2014). Short-term weight loss seems to be comparable between LSG and RYGB (Sjostrom et al., 2012; Boido et al., 2015; Cho et al., 2015), but long term data seems to be in favor of RYGB from the weight loss as well as from the T2DM resolution stand point (O’Brien, 2015). Medications for T2DM and hypertension were decrease or withdrawn for those undergoing bariatric surgery, as early as prior to hospital discharge (Tritsch et al., 2015). In line with this, Ching et al. (2016) found a 60% remission rate of T2DM after LSG that did not correlate with weight loss.

Weight loss and improved glycemic profile following LSG have been attributed to a theory denominating the “gastric hypothesis,” which asserts that alterations in the secretion/action of gut hormones such as GLP-1, GIP, leptin, and PYY triggered by direct stomach manipulation are responsible for the rapid restoration of insulin secretion and sensitivity (Yousseif et al., 2014). Ghrelin, released by the gastric fundus, which is normally excised during LSG (de Oliveira et al., 2015; Yang et al., 2015). Thus, decreased levels of ghrelin were also proposed to be one of the mechanisms that result in metabolic benefits following LSG. However, similar weight loss and improved glucose profile following LSG were found in ghrelin-deficient mice that was genetically modified compared to that in wild type mice (Chambers et al., 2013), suggesting that decreased ghrelin is not a critical factor in T2DM remission (Ching et al., 2016).

In summary, glucose regulation after LSG seems to be partially medicated by the changes in gut hormones post-surgery and partially by the weight loss itself. The role of parasympathetic nervous system in regulating glucose homeostasis post-sleeve gastrectomy is less defined or data is lacking at least. It is more likely; however, that the parasympathetic nervous system is more involved (directly or indirectly) in changes in food intake behavior after LSG.

## Conclusion

Bariatric surgery achieves sustainable improvements in treating metabolic dysfunction related to obesity and improves overall health. The underlying mechanisms by which these procedures cause weight loss and metabolic improvement appear to be diverse and are not yet fully identified. Current evidence suggests that the autonomic parasympathetic nervous system (mainly through its vagus arm) contributes to food intake reduction and improvements in glucose homeostasis following bariatric surgeries. The sympathetic nervous system is also altered after bariatric surgery, with human models showing decrease in systemic tone to the that might correlates with improvement in blood pressure and other homeostatic patterns. Rodent models of RYGB suggest a selective increase in the splanchnic sympathetic nerve activity of the gut, innervating the visceral fat and leading to augmented sympathetic-mediated thermogenesis. Future studies that further unfold details about the underlying molecular mechanisms communicating new energy signals from the gut to the brain along a neuro-hormonal pathway is essential to help us understand how this procedure induces its powerful metabolic effects. Development of effective and less-invasive therapies for weight-management (being surgical or pharmacological) would highly benefit from this information.

## Author Contributions

MM conceptualized the idea and design of the review and critically revised the manuscript. ZA and HW wrote the first draft of the manuscript. All authors contributed to the article and approved the submitted version.

## Conflict of Interest

The authors declare that the research was conducted in the absence of any commercial or financial relationships that could be construed as a potential conflict of interest.

## Publisher’s Note

All claims expressed in this article are solely those of the authors and do not necessarily represent those of their affiliated organizations, or those of the publisher, the editors and the reviewers. Any product that may be evaluated in this article, or claim that may be made by its manufacturer, is not guaranteed or endorsed by the publisher.
